# Effect of droperidol addition to fentanyl-based intravenous patient-controlled analgesia on postoperative nausea and vomiting: a single-center retrospective cohort study

**DOI:** 10.1186/s40981-020-00396-7

**Published:** 2020-11-09

**Authors:** Shuji Uda, Chikashi Takeda, Toshiyuki Mizota

**Affiliations:** Department of Anesthesia, Kyoto University Hospital, 54 Shogoin-Kawahara-Cho, Sakyo-Ku, Kyoto, 606-8507 Japan

To the editor,

Droperidol addition to morphine-based intravenous patient-controlled analgesia (ivPCA) decreased postoperative nausea and vomiting (PONV) in some studies [1–4]; however, the effect of droperidol addition to fentanyl-based ivPCA on PONV is unclear. The antiemetic effect of droperidol addition and its optimal dose may be different between morphine-based and fentanyl-based ivPCA, because fentanyl-based ivPCA, unlike morphine-based ivPCA, is often accompanied by baseline infusion. The only study which showed an association between droperidol addition to fentanyl-based ivPCA and decreased PONV did not include patients undergoing body cavity surgeries [5]. Because surgery type has been suggested to affect PONV risk [6], the antiemetic effect of droperidol addition to ivPCA might differ among surgery type. Therefore, we investigated the effect of droperidol addition to fentanyl-based ivPCA on the development of PONV in patients who underwent gynecologic laparoscopic surgery.

This single-center, retrospective study was approved by the ethics committee of Kyoto University Hospital (approval number R1272-3; January 23, 2020). Patients who underwent laparoscopic gynecological surgery (adnexal surgery and/or hysterectomy) under general anesthesia at Kyoto University Hospital between 2012 and 2018 and who received fentanyl-based ivPCA were included. The exposure of interest was droperidol addition to fentanyl-based ivPCA. We used a disposable patient-controlled anesthesia (PCA) device which delivers 1 ml bolus with a lockout interval of 10 min, combined with a baseline infusion of 1 ml/h. The primary outcome measure was the incidence of PONV, defined as the occurrence of at least one episode of nausea or vomiting during the 2-day postoperative period. We conducted a multivariable logistic regression analysis to assess the independent relationship between droperidol addition to ivPCA and PONV. We selected six potentially confounding variables (age, smoking history, duration of surgery, anesthetic method, intraoperative antiemetic use, and the concentration of fentanyl to be filled into the PCA device) based on clinical relevance and the available literature [6–9]. Two-sided *P* < 0.05 was considered to be statistically significant.

Among 588 study participants, droperidol was added to ivPCA in 350 patients (60%; Table 1). The median concentration of droperidol to be filled into ivPCA was 50 μg/mL (range 20–167 μg/mL). PONV occurred in 56% and 76% of patients with or without droperidol via ivPCA, respectively (*P* < 0.001). Multivariable logistic regression analysis revealed that droperidol addition to fentanyl-based ivPCA was independently associated with decreased PONV (adjusted odds ratio 0.383, 95% confidence interval 0.260–0.563, *P* < 0.001; Table 2). The extrapyramidal symptoms were present in 0.9% of the patients who received droperidol via ivPCA.

**Table 1:** Patient characteristics and operative variables according to droperidol use

	All patients (*n* = 588)	Without droperidol (*n* = 238)	With droperidol (*n* = 350)	*P* value
Age (years)	46 [38–58]	49 [40–61]	45 [37–55]	< 0.001
BMI (kg/m^2^)	21.5 [19.5–24.2]	21.5 [19.5–24.3]	21.6 [19.5–23.8]	0.794
Ever smoker	147 (25%)	66 (28%)	81 (23%)	0.207
ASA-PS (1/1E/2/2E/3/missing)	301/8/263/3/11/2	99/2/125/1/9/2	202/6/138/2/2/0	< 0.001
Malignancy	189 (32%)	69 (29%)	120 (34%)	0.177
Duration of surgery (min)	212 [147–312]	202 [152–289]	214 [147–322]	0.417
Concentration of fentanyl to be filled into ivPCA (μg/mL)	25 [20–25]	25 [20–25]	20 [20–25]	0.108
Total intraoperative fentanyl dose (μg)	250 [150–300]	250 [150–300]	250 [150–300]	0.380
Intraoperative antiemetic use	275 (47%)	80 (34%)	195 (56%)	< 0.001
Blood loss (mL)	25 [0–110]	25 [0–110]	25 [0–104]	0.973
Total intravenous anesthesia	95 (16%)	45 (19%)	50 (14%)	0.135

Continuous variables were presented as medians [interquartile range] and compared using the Mann-Whitney *U* test. Categorical variables were presented as numbers (percentage) and compared using the Pearson chi-square test or Fisher exact test, as appropriate. *BMI* body mass index, *ASA-PS* American Society of Anesthesiologists physical status classification, *ivPCA* intravenous patient-controlled analgesia

**Table 2:** Multivariable logistic regression analysis assessing independent association between addition of droperidol to ivPCA and PONV

Variable	Adjusted OR (95% CI)	*P* value
Addition of droperidol to ivPCA	0.383 (0.260–0.563)	< 0.001
Age (per 1 year)	0.991 (0.977–1.003)	0.165
Ever smoker	0.600 (0.403–0.895)	0.012
Duration of surgery (per min)	1.000 (0.999–1.001)	0.833
Intraoperative antiemetic use	0.976 (0.667–1.428)	0.899
Concentration of fentanyl to be filled into ivPCA (per μg/mL)	0.960 (0.908–1.015)	0.152
Total intravenous anesthesia	0.513 (0.316–0.831)	0.007

*ivPCA* intravenous patient-controlled analgesia, *PONV* postoperative nausea and vomiting

Our study included patients who underwent laparoscopic gynecological surgery, which was not included in the previous study [5] and still showed a significant association between droperidol administration via ivPCA and a reduction in PONV incidence. Based on the results of our study, the extrapyramidal symptoms are considered a rare problem in the patients who received droperidol via ivPCA. In conclusion, droperidol addition to fentanyl-based ivPCA may reduce PONV in patients who underwent laparoscopic gynecological surgery.

## Data Availability

The datasets used and analyzed in this study are available from the corresponding author on reasonable request.

## Abbreviations

ivPCA: Intravenous patient-controlled analgesia
PCA: Patient-controlled anesthesia
PONV: Postoperative nausea and vomiting

## References

[1] Kuo YM, Tsou MY, Chang WK, Chan KH, Chang KY (2012). To add or not to add? An empirical study on droperidol and intravenous patient-controlled analgesia. J Chin Med Assoc.

[2] Klahsen AJ, O'Reilly D, McBride J, Ballantyne M, Parlow JL (1996). Reduction of post-operative nausea and vomiting with the combination of morphine and droperidol in patient-controlled analgesia. Can J Anaesth.

[3] Barrow PM, Hughes DG, Redfern N, Urie J (1994). Influence of droperidol on nausea and vomiting during patient-controlled analgesia. Br J Anaesth.

[4] Sharma SK, Davies MW (1993). Patient-controlled analgesia with a mixture of morphine and droperidol. Br J Anaesth.

[5] Hirata I, Iwamoto M, Matsui H, Yoshinuma H, Funakoshi R (2020). Droperidol reduces postoperative nausea and vomiting and supports the continuation of intravenous patient-tontrolled analgesia with fentanyl. J Pharm Pharm Sci.

[6] Gan TJ, Belani KG, Bergese S, Chung F, Diemunsch P, Habib AS, Jin Z, Kovac AL, Meyer TA, Urman RD, Apfel CC, Ayad S, Beagley L, Candiotti K, Englesakis M, Hedrick TL, Kranke P, Lee S, Lipman D, Minkowitz HS, Morton J, Philip BK (2020). Fourth consensus guidelines for the management of postoperative nausea and vomiting. Anesth Analg.

[7] De Oliveira GS, Jr., Castro-Alves LJ, Ahmad S, Kendall MC, McCarthy RJ. (2013). Dexamethasone to prevent postoperative nausea and vomiting: an updated meta-analysis of randomized controlled trials. Anesth Analg.

[8] De Oliveira GS, Jr., Castro-Alves LJ, Chang R, Yaghmour E, McCarthy RJ. (2012). Systemic metoclopramide to prevent postoperative nausea and vomiting: a meta-analysis without Fujii's studies. Br J Anaesth.

[9] Roberts GW, Bekker TB, Carlsen HH, Moffatt CH, Slattery PJ, McClure AF (2005). Postoperative nausea and vomiting are strongly influenced by postoperative opioid use in a dose-related manner. Anesth Analg.

